# Establishing the values for patient engagement (PE) in health-related quality of life (HRQoL) research: an international, multiple-stakeholder perspective

**DOI:** 10.1007/s11136-016-1465-5

**Published:** 2016-12-08

**Authors:** Kirstie Haywood, Anne Lyddiatt, Samantha J. Brace-McDonnell, Sophie Staniszewska, Sam Salek

**Affiliations:** 1 Royal College of Nursing Research Institute, Department of Health Sciences, Warwick Medical School, University of Warwick, Coventry, UK; 2 Ingersoll, ON Canada; 3 Warwick Clinical Trials Unit, University of Warwick, Coventry, UK; 4 Independent Cancer Patient Voice (Reg Charity no. 1138456), London, UK; 5 The School of Life and Medical Sciences, University of Hertfordshire, Hatfield, UK

**Keywords:** Patient involvement, Patient engagement, Values, HRQoL, Patient-reported outcomes

## Abstract

**Purpose:**

Active patient engagement is increasingly viewed as essential to ensuring that patient-driven perspectives are considered throughout the research process. However, guidance for patient engagement (PE) in HRQoL research does not exist, the evidence-base for practice is limited, and we know relatively little about underpinning values that can impact on PE practice. This is the first study to explore the values that should underpin PE in contemporary HRQoL research to help inform future good practice guidance.

**Methods:**

A modified ‘World Café’ was hosted as a collaborative activity between patient partners, clinicians and researchers: self-nominated conference delegates participated in group discussions to explore values associated with the conduct and consequences of PE. Values were captured via post-it notes and by nominated note-takers. Data were thematically analysed: emergent themes were coded and agreement checked. Association between emergent themes, values and the Public Involvement Impact Assessment Framework were explored.

**Results:**

Eighty participants, including 12 patient partners, participated in the 90-min event. Three core values were defined: (1) building relationships; (2) improving research quality and impact; and (3) developing best practice. Participants valued the importance of building genuine, collaborative and deliberative relationships—underpinned by honesty, respect, co-learning and equity—and the impact of effective PE on research quality and relevance.

**Conclusions:**

An explicit statement of values seeks to align all stakeholders on the purpose, practice and credibility of PE activities. An innovative, flexible and transparent research environment was valued as essential to developing a trustworthy evidence-base with which to underpin future guidance for good PE practice.

## Introduction

Patient engagement (PE), or patient and public involvement (PPI), is increasingly viewed as a cornerstone of health-related research activities and policy making [1]. Effective patient engagement (PE) can profoundly change how patient-centred research is conceptualised and conducted, resulting in better patient-centred care, management and measurement [2–4]. However, these partnerships require new skills and sustained efforts for all stakeholders: understanding the values that different stakeholders aspire to provide an essential foundation for effective PE.

The values associated with good PE in health and social care research have recently been defined as ‘the established collective moral principles and accepted standards of a person or a social group; principles, standards or qualities considered worthwhile or desirable’ [5]. In supporting patients and health professionals to participate in effective PE, an agreed statement of values endeavours to support everyone in understanding their role—why we do it, what is important, and to whom. Experience has shown that different stakeholders often hold different values associated with the practise of PE; such discrepancies in values can result in conflict and a failure in the conduct of effective PE and its likely impact [5, 6]. In developing our understanding of the diversity in values, we can acknowledge and understand them and work within a framework that considers different perspectives, different motivations, and recognises the potential for conflict to emerge when such diversity exists. Developing strategies for managing such potential conflict are essential, highlighting the importance of understanding values at the outset of a programme of research and identifying common values that everyone recognises as well as respecting the diversity of values that may be present in a research team. A consensus process involving members of the Health Technology Assessment International (HTAi) Patient and Citizen Involvement Group (PCIG) recently defined five core values and standards to capture common understanding with which to underpin effective PE in HTAi processes: (1) relevance, (2) fairness, (3) equity, (4) legitimacy and (5) capacity building (http://www.htai.org/interest-groups/patient-and-citizen-involvement/pcig-home/values-and-standards.html) [7]. However, such explicit value statements for PE are rarely stated [5] and have not been explored for health-related quality of life (HRQoL) research.

The active engagement of patients as research partners is increasingly viewed as essential to ensuring that the patient perspective is considered throughout the research and healthcare process that research focuses on issues of importance to patients and that research waste is avoided [2, 3, 8, 9]. However, guidance for active PE in HRQoL research does not exist, and the evidence-base is limited, primarily because of poor reporting [10]. Moreover, the underpinning philosophical values held by different stakeholders may affect the PE approaches adopted and its likely impact. The results of the first International Society of Quality of Life (ISOQOL) research PE World Café [4] highlighted the need to agree and promote best practice for PE in patient-reported outcomes (PRO) and HRQoL research that is suitable in different healthcare and political systems. This can be achieved through the creation of a set of values and high level principles to support best practice, developed with wide stakeholder engagement and in a scientifically robust manner to ensure use in practice [5, 10, 11].

As a continuation of ISOQOL’s commitment to embracing PE in measurement science and HRQoL research, in October 2014 it hosted a second PE World Café with the intent to develop clarity and understanding of PE/PPI with an international audience of patients, healthcare professionals and researchers. It was envisaged that this would lead to an international consensus on a set of values and quality standard statements for PE/PPI in the development and use of HRQoL measurement.

## Methods

### Setting

This exploratory research exercise was part of the 21st annual ISOQOL conference, held in Berlin, using the international attendees as participants. Past experience highlighted the importance of engaging with patients as partners throughout the planning and execution of the research [4]. The outcome of the first ‘PE Café’ held in 2013 identified the need for active collaboration with patient partners. As a consequence, ISOQOL awarded the first two Patient Engagement Scholarships during 2014.

The PE Scholars [SBM, AL] worked collaboratively with the co-chairs of the PE special interest group (SIG) [KH, SS] in the development, conduct and analysis of the second PE Café, with the aim of ensuring that issues of importance to patients were considered throughout all stages of the initiative. Agreement on key decisions, methods and analyses was required between all four members.

### Participants

Informal invitations were sent to all conference attendees and the ISOQOL membership consisting of researchers, healthcare professionals and academics with a common interest in measurement science, HRQoL and PRO-related research from across the wide spectrum of healthcare, health service research and industry. Historically, ISOQOL conferences have not witnessed the attendance of large numbers of patients [4]. However, fuelled by growing interest in PE and support from ISOQOL, there were 12 patient partners present during the conference, all of whom were invited to participate in the event. We sought to establish a collaborative partnership between all participants, underpinned by the intention to co-produce values that should underpin PE in HRQoL research.

Participants self-selected to attend the event. Traditionally, attendees are free to move between on-going parallel sessions. However, due to the need for continuity in discussion, we encouraged all attendees to participate in each ‘course’ of the event.

### The PE cafe

A modified ‘World Café’ [12] (http://www.theworldcafe.com/method.html) was hosted as a collaborative activity between patient partners, clinicians and researchers: self-nominated delegates participated in small and large group discussions to explore values associated with the conduct and consequences of PE in HRQoL research.

**Fig. 1:**
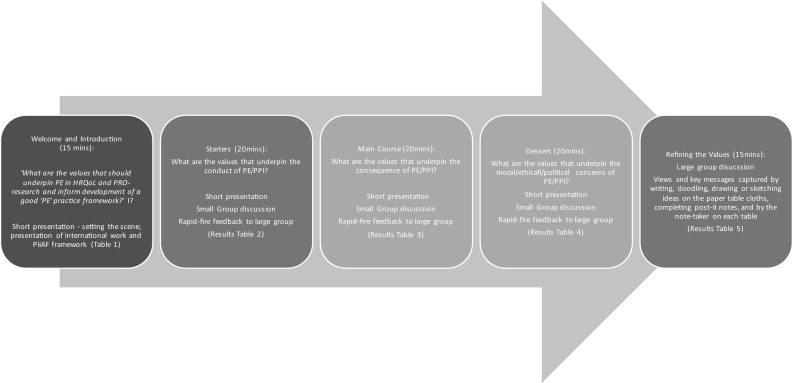
Key Steps in Patient Engagement Café—Establishing the values associated with PE in HRQoL research

The format of a ‘World Café’ supports the exploration of new knowledge and views through interactive small group discussions [4, 12]. Traditionally, World Café events may take several hours to explore the concept of interest, with participants moving between tables and developing discussions with other participants [12]. However, due to conducting the event within the ISOQOL conference, the process was modified to fit into a 90-min ‘symposium’ session; such a modification has been successfully described before [4]. The key steps of the event are summarised in Fig. 1. On arrival at the event, participants were encouraged to sit at small round tables (up to 10 per table) with other guests with whom they were not previously conversant. In discussion with the PE Scholars, it was decided that all 12 patient participants would form a single table group. This was driven by the desire to understand whether patients valued participation in research differently than other symposium participants.

The overall theme of the event was ‘What are the values that should underpin PE in HRQoL and PRO-research and inform development of a good ‘PE’ practice framework?’ To achieve this, reference was made to the Public Involvement Impact Assessment Framework (PiiAF) which provides a typology of explicit values underpinning PE/PPI in health and social care research ([5, 6]; http://piiaf.org.uk/]. This typology describes three broad value systems and 15 associated value clusters (Table 1) and supported the exploration of three ‘menu’ questions. The first course (‘Starters’) asked: What are the values that underpin the conduct of PE/PPI? This question sought to explore the values associated with the process of or ‘doing PE/PPI’, encompassing mutuality, reciprocity, reflexivity and learning from each other. The second course asked: What are the values that underpin the consequence of PE/PPI? This question sought to explore the substantive values or impact of engaging with PE/PPI, for example, enhancement of the quality and relevance of research. Finally, the third course (‘Dessert’) asked the question: ‘What are the values that underpin the moral, ethical and/or political concerns of PE/PPI?’ This question sought to explore the normative values associated with the conduct of PE/PPI such as valuing an individual’s rights and issues of empowerment.

**Table 1:** Public Involvement Impact Assessment Framework (PiiAF) Overview of values [5]

Process-related—conduct	Substantive—consequences	Normative—moral, ethical, political
*Partnership/equality* Relationship based upon sharing power and decisions in equal, reciprocal and collaborative PI processes	*Effectiveness* PI has an effect in research and implementation	*Empowerment* Transfer of control, self-help, seeking to overcome discrimination and oppression
*Respect/trust* Respecting diversity, values, skills, knowledge and experience in mutually beneficial PI processes	*Quality/relevance* Increasing the quality, relevance appropriateness and credibility of research through PI	*Rights* Refers to PI being of intrinsic value and the fundamental human right to have a say
*Openness/honesty/flexibility/commitment* Processes and attitudes being open, honest, flexible, and committed to PI	*Validity/reliability* Processing reliable, valid and rigorous knowledge. Recognises the beneficial impact of PI	*Change/action* The idea of generating or translating knowledge into action in order to bring about change
*Independence* Research teams achieving their objectives away from managerial control; in research team interactions through autonomous voices and actions	*Representativeness/Objectivity/Generalizability* Representative, objective, and generalizable knowledge through PI	*Accountability/transparency* PI involves clarifying the relationship between the research and wider society: ‘Noting about me without me’
*Clarity* Purpose, processes, communication, and definition of PPI to all	*Evidence-base* Generating a substantial and rigorous evidence-base about PI	*Ethical values* Ethical awareness in order to protect from harm

*PI* Public Involvement

To set the scene, an overview of international work undertaken by a range of groups exploring ‘values’ associated with good PE was presented, for example, the activities of the Health Technology Assessment international (HTAi) Patient and Citizen Involvement Group (PCIG) [7] (http://www.htai.org/interest-groups/patient-and-citizen-involvement/pcig-home/values-and-standards.html) and the Outcome Measures in Rheumatology (OMERACT) initiative [2, 13] (http://www.omeract.org). Each course was subsequently introduced with a short presentation that sought to highlight the value clusters described within each of the three PiiAF value systems.

Participants had up to 10 min to discuss the key question(s) posed during each ‘course’ and were encouraged to consider the range of values associated with PE and how these could underpin a ‘vision’ for good PE in HRQoL and PRO-related research. Each table provided a rapid-fire feedback of salient points at the end of each course. Menu cards on each table listed the ‘menu’ questions and the definitions included within the PiiAF framework.

A café event views everyone as equal with all contributions judged to be valid [12]. Participants were encouraged to contribute their views and values, whilst being open to the views and values of others. Views and any key messages arising during the group discussions were captured by writing, doodling, drawing or sketching ideas on the paper tablecloths or completing post-it notes. The use of different methods for capturing data seeks to enhance creative thinking, expression and communication [4, 12, 14]. The ‘PE Theme Team’ (KH, SS, SBM, AL) acted as facilitators and supporters for participants, providing clarification and a ‘light touch’ direction as and when requested. In addition, each table nominated a ‘table host’ whose role was to keep a focus on the question and encourage participation from all participants, and a rapporteur whose role was to keep summary notes of the table discussion; they were also required to feedback the top three ‘values’ to the room. Conversations were further facilitated by the provision of confectionary and water. Following each course post-it notes were collected by café facilitators and displayed on the ‘Wall of Engagement’—a large A0-sized poster displaying the typology of values. Participants were encouraged to add to this poster during the café and throughout the conference.

### Analysis

An initial meeting (KH, SS, SBM) was conducted to examine the content of the data collected and identify initial concepts across the different forms of data collected. Data in the form of key phrases, statements, lists, sketched ideas and drawings were independently extracted from the accumulated post-it notes, detailed table notes and doodles. A thematic analysis was undertaken where two members of the core team (KH, SS) familiarised themselves with the different forms of data and added initial codes [15]. Constant comparison across the different forms of data informed an initial thematic framework to enable consistent coding of the data. If themes were identified from the data that did not fit the initial coding framework, a new code was established to involve the theme in the analysis. The researchers (KH, SS) worked independently to identify themes, but met to discuss the themes and establish consensus. All themes, particularly where consensus could not be achieved, were further discussed and agreed with the PE Scholars (SBM, AL). This enabled analysis codes to be modified as new ideas were developed [15]. All members of the core research team (KH, SS, SBM, AL) then commented on the proposed themes and supporting evidence. Reliability was therefore established through discussion, and findings were based on researcher agreement [16, 17]. Once the thematic analysis was complete, the association between the data, emergent themes and the PiiAF framework was explored and mapped.

## Results

### Socio-demographics

A total of 524 people were registered conference attendees; the majority were ISOQOL members (*n* = 298). There were 12 registered patient partners, including the two PE Scholars (SBM, AL). Most participants were from North America (USA *n* = 171; Canada *n* = 37) or Europe (UK *n* = 79; Germany *n* = 45, The Netherlands *n* = 32, France *n* = 29, Norway *n* = 22; Sweden *n* = 13). Where provided, most participants described their discipline as academic and/clinical (82%).

Eighty participants, including the 12 patient partners, engaged in the 90-min café event. Three of the patient participants were from North America and the remaining nine from Europe (UK *n* = 4; The Netherlands *n* = 2; Germany *n* = 1; France *n* = 1; Switzerland *n* = 1). These patients represented various conditions including rheumatology, respirology, oncology and haematological malignancies. One patient partner was an informal carer. Four of the patient partners had undergone specific training in their role as patient partners; two were graduates of Canada’s Patient and Community Engagement Research Programme (PACER) (https://obrieniph.ucalgary.ca/pacer), and two were long-term members of the OMERACT initiative (including AL). The remaining patients were relatively new to their role as patient partners and had received varying levels of training in this role.

The initial grouping of participants with patients forming a single group at a separate table soon became untenable due to an overwhelming desire of all participants to be fully integrated across tables. This was responded to immediately, and one to two patient partners were invited to join each round table. This unique experience attracted positive comment from the floor:

It took [another organisation] 2-years to realise that patient partners should be integrated and not kept at a separate table – but it took ISOOQL just 20 min!

### Emerging themes

The world café format facilitated a dynamic, inspiring and often thought-provoking debate between patient partners, researchers and healthcare professionals. The resultant wealth of data explored the many values and challenges associated with PE in HRQoL and PRO research. Initial categories were developed deductively per the three key questions posed during the exercise—with a focus on the process, substantive and normative values associated with PE. The results from each question are summarised and presented in Tables 2, 3 and 4, respectively.

**Table 2:** Values associated with the process (conduct of, or doing) PE in HRQoL research

1.1 Partnership/equality	1.2 Respect/trust	1.3 Openness/honesty	1.4 Independence	1.5 Clarity
*Genuine relationship* Mutual respect (skills, knowledge, contribution) Effective collaboration Defined/transparent roles Challenges associated with PE: Equality Burden Defining approaches to PE: what works, for whom, when and in what context? Defining roles Mutual respect	*Importance of building relationships* Mutual respect Listening to understand Different values Different skills Different approaches to PE: what works, for whom, when and in what context? Interest in ‘how to do PE’ and the challenges associated: Resolve conflict Burden Defining roles Mutual respect	*Improve quality of research* Greater transparency Clarity in purpose and processes Respectful of different viewpoints/new insights Relationship building—co-learning Interest in ‘how to do PE’ and the associated challenges: Resolve conflict Burden Defining roles Mutual respect Need for flexibility/willingness to change	??	*Improving the quality of research* Transparency Clarity in purpose and processes Research more explicit Asking the ‘right’/obvious questions Enhance validity Appreciative of the challenges associated with PE: Poor evidence-base Different approaches to PE: what works, for whom, when and in what context?
*Overriding themes: What is valued about doing PE?* Effective collaborative relationships are underpinned by mutual respect for different values and skills Effective partnership can improve the quality of research = consequence (substantive value) However, the challenges of doing good PE are recognised This recognition underpins the importance of developing a strong evidence-base for good PE to inform good practice guidance: what works, for whom, when and in what context				
*VALUE* developing a genuine relationship between all stakeholders—a collaborative, respectful, deliberative and transparent relationship based on trust and mutual respect/reciprocity *REQUIRES* work towards developing a genuine, honest and open relationship between all stakeholders *REQUIRES* guidance reapproaches to PE, an awareness of the challenges and how to resolve

**Table 3:** Values associated with the substantive impact (consequence) of doing PE in HRQoL research

2.1 Effectiveness	2.2 Quality/relevance	2.3 Validity/reliability	2.4 Representativeness/objectivity/generalisability	2.5 Evidence-base
*Improve the quality, dissemination and impact of research* Transparency Respectful of different viewpoints/new insights Research more explicit Impact *Underpinned by the importance of developing an evidence-base of effectiveness* Different approaches to PE: what works, for whom, when and in what context? Mutual respect	*Improve the quality of research* Transparency Respectful of different viewpoints/new insights Research more explicit Impact Improves the relevance and credibility of research to patients’ needs (reality check) *Underpinned by—the importance of building relationships* Mutual respect Listening to understand Different values Different skills Different approaches to PE: what works, for whom, when and in what context? *Underpinned by—the challenge and importance of developing an evidence-base of effectiveness* Different approaches to PE: what works, for whom, when and in what context?	*Improve the validity, relevance, credibility and quality of research* Transparency Respectful of different viewpoints/new insights Research more explicit Impact Improves the relevance and credibility of research to patients’ needs (reality check) *Underpinned by—the importance of building relationships* Mutual respect Listening to understand Different values Different skills Different approaches to PE: what works, for whom, when and in what context? *Underpinned by—the challenge and importance of developing an evidence-base of effectiveness* Different approaches to PE: what works, for whom, when and in what context?	What level of PE representativeness is meaningful and appropriate? Lack of clarity Lack of guidance	*Challenge and Importance of developing an evidence-base of effectiveness* Different approaches to PE: what works, for whom, when and in what context?
*Overriding themes: What are the consequences of PE?* Doing PE improves the quality, validity, relevance and credibility of research Doing PE improves the dissemination and impact of PE These benefits are generated from strong, effective relationships underpinned by mutual respect and a valuing of difference skills and values. However, guidance for the level of PE representativeness that may be viewed as meaningful and appropriate is required Moreover, the evidence-base for this impact is limited, and greater efforts are required to develop a strong evidence-base: what to do, when, with whom and in what context				
*VALUE* the potential impact of PPI on enhancing the quality, relevance and credibility of research *VALUES* the need for a creative and innovative research environment which values high quality, consistent and rigorous research and methods to underpin approaches to PPI and hence inform a strong evidence-base *REQUIRES* work towards developing a genuine, honest and open relationship between all stakeholders *REQUIRES* guidance reapproaches to PE and awareness of challenges (and how to resolve)

**Table 4:** Values associated with the normative concerns (moral, ethical and political concerns) underpinning PE in HRQoL research

3.1 Empowerment	3.2 Rights	3.3 Change/action	3.4 Accountability/transparency	3.5 Ethical values
*Values to need to establish a genuine relationship to ensure effective PE* Mutual respect (skills, knowledge, contribution) Effective collaboration Defined/transparent roles Diversity of views/seek to understand diverse needs and values *Challenges associated with empowerment and PE* Equality Burden Defining approaches to PE Defining roles/clarity Mutual respect Diversity	*Value the rights of patients to contribute to the research process* their fundamental right to have a say. Requires a process to enable effective involvement/PE *Underpinned by: the importance of building relationships* Mutual respect Listening to understand Different values Different skills Enabling patients to contribute Different approaches to effective PE: what works, for whom, when and in what context? *Underpinned by the challenge and Importance of developing an evidence-base of effectiveness* Different approaches to PE: what works, for whom, when and in what context?			
*Overriding themes: moral, ethical, political concerns* The fundamental right of the patient to have a say and to be empowered in their contribution to the research process was widely valued. However, it was recognised that this requires the establishment of a genuine relationship between patient and other research partners, underpinned by mutual respect, clarity in roles to be undertaken, and valuing of different views and perspectives. An awareness of the different approaches to PE—and what works, for whom, when and in what context—was considered essential to enabling effective involvement and requires the development of a strong evidence-base with which to inform good practice guidance.				
*VALUE* developing a genuine relationship (based on mutual respect, transparency and collaboration) underpinned by understanding the diverse needs, views and values of patients *VALUE* the rights of patients to contribute to the research process and the processes to enable effective involvement

1. Values underpinning the conduct of PE
  With respect to the values that may underpin the process (conduct of, or ‘doing’) of PE, the overarching theme was the importance of effective collaborative relationships underpinned by several key sub-themes: the importance of mutual respect for differing values and skills, greater transparency and the need for clarity in purpose and process (Table 2). Participants did not explore issues of independence included in the PiiAF framework. Illustrative quotes include:
  Trust is something that grows as the research develops; trust is more of an outcome – [it’s] important to build an environment where patients can trust
  Don’t need to agree with the patient, but do need to debate and discuss
  Partnership negotiation depends on nature of involvement
2. Values underpinning the consequences of PE
  Two overarching themes emerged following an exploration of the substantive values (consequences) associated with PE: firstly, the impact of PE on the quality, relevance and credibility of the research; and second, the challenges and importance of developing an evidence-base for PE practice (Table 3). The importance of developing effective relationships between all stakeholders was central to both themes (Table 3). Illustrative quotes include:
  Effectiveness is a shared value; it is important that patient partners are involved in defining what impact will look like
  We collect data from preconceived medical ideas; we need data from patients to know what to measure
3. Values underpinning the moral, ethical and/or political concerns of PE
  With respect to the normative concerns (moral, ethical and/or political) explored in association with PE, the fundamental right of the patient to have a say and to be empowered in their contribution to the research process was widely valued (Table 4). However, it was recognised that this requires the establishment of a genuine relationship between the patient(s) and other research partners, underpinned by mutual respect, clarity in roles to be undertaken, and valuing of different views and perspectives. An awareness of the different approaches to PE—and what works, for whom, when and in what context—was considered essential to enabling effective involvement and requires the development of a strong evidence-base with which to inform good practice guidance. Illustrative quotes include:
  My job as a patient is not to tell my story – it is to bring a reflective voice to the table. But this is not all that patients can offer
  Important to know what people are involved and what they wish to achieve
  ISOQOL should commit more to diversity – involving different people with barriers to participation. Not just getting the right patients that are articulate, educated and often, white middle-class
  Patients as researchers also need to be ethical in working with other patients
  Subsequently, an inductive approach was undertaken with emergent themes informed by data generated through discussion within these three categories. Whilst it was possible to categorise the raw data per the PiiAF framework (both the value systems and associated value clusters), the language adopted by the participants did not always map readily to the language of the framework. Rather, it drove the development of new emergent themes and extensive sub-themes—which are discussed below and illustrated in Table 5. In the final distillation, six overriding themes, with several key sub-themes were defined: (1) building genuine relationships between all stakeholders; (2) challenges associated with effective PE; (3) improving the quality, relevance and credibility of research; (4) improving the dissemination, implementation and impact of research; (5) different approaches to PE; and (6) the Importance of developing a strong evidence-base for PE practice (Table 5).

**Table 5:** Overriding themes and core values associated with PE in HRQoL Research and mapping to the PiiAF framework [6]

Themes	Values	Mapping to PiiAF
1. Building genuine relationships between all stakeholders Built on mutual respect for differing skills, values and knowledge Listening to understand: co-learning Valuing diversity Effective collaboration: honesty, openness. listening to understand Defining/transparent roles: a partnership throughout the research process	1. Building relationships Developing a genuine relationship between all stakeholders A collaborative, respectful, deliberative and transparent relationship based on trust, reciprocity, co-learning and mutual respect	*Conduct of PPI* Partners hips/equality: equal, reciprocal and collaborative Respect/trust: diversity, values, skills, knowledge and experience Openness/honesty/flexibility Commitment: processes and attitudes Clarity: purposes, processes, communication and definition of PPI ‘What’s important?’ *Add Normative Value*—*fundamental right to have a say!*
2. Challenges associated with effective PPI Equality Burden Defining roles Resolving conflict Poor/limited evidence-base		
3. Improving the quality, relevance and credibility of research Transparency Clarity in purpose and process New/unique insights: experiential knowledge of patients Research more explicit Asking the ‘right’ questions Enhanced validity: improved relevance and credibility of research to patients’ needs	2. Improving research quality, relevance and implementation The potential for effective PE to enhance the quality, relevance, credibility and implementation of research	*Consequences of PPI* Effectiveness: an effect in research and implementation Quality/relevance: quality, relevance, appropriateness and credibility of research Validity/reliability: processing reliable, valid and rigorous knowledge ‘Why do we do it?’
4. Improve the dissemination, implementation and impact of research		
5. Different approaches to PPI: What works for whom, when and in what context What level of representativeness is meaningful and appropriate ‘Not just the posh articulate’	3. Developing best practice A creative and innovative research environment which values high quality, consistent and rigorous research and methods to underpin approaches to PPI, and hence inform a developing evidence-base	Consequences of PPI Evidence-base: a substantial and rigorous evidence-base ‘Why do we do it?’
6. Importance of developing the evidence-base ‘How to do effective PPI?’ Challenges

These themes underpin three core values:

1. *Building relationships* developing a collaborative, respectful, deliberative and transparent relationship based on trust, reciprocity, co-learning and mutual respect. This requires guidance for effective PE/PPI practice and a developing evidence-base; that is, what works, for whom, when and in what context.
2. *Improving research quality and impact* the potential for effective PE to enhance the quality, relevance, credibility and implementation of research. This requires a genuine, honest and open relationship between all stakeholders underpinned by clear guidance for approaches towards effective PE/PPI.
3. *Developing best practice* a creative and innovative research environment which values high quality, consistent and rigorous research and methods to underpin approaches to PE/PPI and thus inform a developing evidence-base. This requires a willingness to embrace the challenges associated with PE/PPI, underpinned by flexibility, honesty and openness.

### Mapping values against the PiiAF Framework

The defined values were finally mapped against the PiiAF framework (Table 5). The first of the values—‘building relationships’, maps onto values associated with the process of PE (that is, what’s important about PE), embracing concepts of partnership/equality, respect/trust, openness/honesty and clarity in purpose and process. This value also embraces a normative value—that is, the fundamental right for all stakeholders to have a say. The second two values—‘improving research quality and impact’ and ‘developing best practice’, mapped onto substantive values (that is, why we do PE), embracing effectiveness, quality and relevance, validity and reliability, and the influence on a developing evidence-base.

## Discussion

This study provides the first international exploration of values that should underpin PE in measurement science, HRQoL and PRO-related research, reflecting the perspective of patients, healthcare professionals, researchers and academics from healthcare, policy and industry. The result is an explicit statement of three core values that seek to align all stakeholders on the purpose, practice and credibility of PE activities. Participants valued the importance of building genuine, collaborative and deliberative relationships between all stakeholders, underpinned by honesty, respect, co-learning and equity. Also valued was the impact of effective PE on the quality, relevance of research, and the implementation of research findings. An innovative, flexible and transparent research environment was also valued as essential to developing a trustworthy evidence-base with which to underpin future guidance for good PE practice.

In seeking to co-produce a value statement for PE, reference was made to the Public Involvement Impact Assessment Framework (PiiAF) [6]. This framework was informed by an extensive programme of research that sought to define the values associated with best practice PE/PPI in health and social care. The framework was provided as an aid to study participants to guide their deliberations. However, the framework received a mixed response. Whilst some participants found it useful, others found it challenging, and choosing not to refer to it. Nevertheless, the PiiAF provided a useful basis to inform the data analysis. Indeed, the three proposed values clearly mapped to two of the PiiAF value systems and most of the associated cluster values: specifically, values associated with the process and consequence of PE/PPI.

Participation from North America and Europe at the ISOQOL 2014 conference was well balanced, and this mix was similarly represented in the PE café event. Although, as expected, the number of health professionals outnumbered those of patient partners, almost 20% of the group were patient partners. This mix enabled between one and two patients per table discussion, facilitating a real conversation between patients and the ISOQOL community and thus enhancing confidence in the external validity of the results. Patient research partners contribute approximately 10% of OMERACT conference participants—a group that, over the last decade, has championed the active engagement of patients as research partners in PRO-related research [2, 13]. Moreover, both the European League Against Rheumatism (EULAR) (http://www.eular.org/pare_patient_research_partners.cfm) [18, 19] and OMERACT [20] have recommended the inclusion of at least two patient partners per research project; this is a recommendation that should be discussed further within the ISOQOL community.

The patient partners represented a diversity of health conditions and experience of engagement as research partners. Although all patients were sponsored to attend the event and hence may have provided a more positive experience of PE, the task of exploring values associated with PE was novel to all participants. Specific data on health professional participants were not captured, with participants self-selecting to participate in the event and thus were likely to represent a mix of ideas and experiences (both positive and negative). In keeping with the ethos behind good patient engagement [18–20], the ‘PE theme team’ sought to facilitate a collaborative approach to the group discussions and grounded in mutual respect for the perspective of others. Hence, an important learning point was the change from having patients participate as a single table to integration across all tables. The original decision was driven by a desire to understand whether patients and health professionals valued PE differently. However, it was clear that the opportunity for discussion amongst all participants, leading to a transparent, co-production of values was more highly valued by participants.

This is the first study to describe the use of a ‘World Café’ type format to explore the values associated with patient engagement in HRQoL research. The format was well received by all participants—particularly once groups were ‘mixed’—who welcomed the opportunity to openly engage with colleagues within a mutually supportive environment. However, participants were set a significant task that, on reflection, would have benefitted from longer periods for discussion and reflection—as reflected in more traditional World Café approaches [12]—than possible within the available 90 min.

In addition to the recent HTAi PCIG values statement, there are helpful similarities between the values proposed by this study and those of several other groups. OMERACT recognises that effective PE strengthens research; it therefore actively promotes the equitable participation and integration of patient research partners with professional researchers throughout all OMERACT projects [13] (http://www.omeract.org/pdf/OMERACT_Handbook.pdf). The European Patients Forum have defined five values to underpin meaningful patient involvement: (1) appropriate patient representation; (2) building on diversity and pooling knowledge; (3) equality, providing an empowering environment; (4) commitment to patient involvement as a positive, value-adding experience; and (5) respect for patients as equal partners [21] (European Patients Forum: The Value+ Toolkit: http://www.eu-patient.eu/globalassets/projects/valueplus/value-toolkit.pdf). These values are also embraced by the Patient Centred Outcomes Research Initiative (PCORI), where the principles of trust, honesty, co-learning, transparency, reciprocal relationships, partnership and respect underpin their stated approach towards patient-centred outcomes research [8] [http://www.pcori.org/about-us]. The importance of building strong relationships between all stakeholders which facilitates the active involvement and contribution of all members is central to most of these shared values and is mirrored in the recommendations from the ISOQOL PE café participants. Moreover, the importance of establishing and maintaining good relationships between researchers and lay representatives has recently emerged as a key aspect of c*ollective action*—the operational work required to enact PPI practices [22]. This was done through regular communication, managing meetings to address power imbalances and providing opportunities for informal engagement. However, there was some evidence that developing good relationships was difficult when PPI was conducted purely through virtual media [22, 23].

Growing evidence suggests that meaningful PE requires an engaged research team with a shared understanding of the collective values and an awareness of the sustained, long-term effort that should underpin PE. However, additional elements that may facilitate more effective PE moving forward are as follows:

*Methodological guidance*—*developing evidence*-*based guidance for how to do it and what works* PE is far more than supporting patient participation in interviews or focus groups. Rather, PE/PPI is a ‘collaborative partnership between patients and researchers, which is often underpinned by the intent to co-produce knowledge’ [10]. In HRQoL research, such active engagement can, for example, support the production of more patient-relevant research questions, enhance study design so that it is more acceptable and appropriate for patients and inform outcome selection so that the outcomes that really matter to patients are the focus of the research and hence avoid the potential for research waste [9]. Moreover, such engagement can support the selection of measures that are both robust and relevant.

As with many new areas of research, the reporting of PE activities is poor. In order to develop a strong evidence-base that benefits future PE research, the transparency and quality of reporting must be improved. Moreover, the involvement of patient partners as co-authors in published research, and the inclusion of a section which communicates the conduct and value of research to a lay, non-academic audience are increasingly recommended (http://www.bmj.com/campaign/patient-partnership). The GRIPP 2 (Good Reporting of Involving Patients and the Public in research) [10] initiative has provided the first international evidence-based, consensus informed checklist for PE (PPI) reporting. The application of such a framework in HRQoL and PRO-related research has yet to be explored: extending GRIPP 2 for HRQoL could support a developing evidence-base through better reporting, facilitating a clearer evaluation of PE. Once methodological guidance is established, a logical and essential next step is to define the measurable standards—the elements of best practice—against which good practice will be assessed.

*Tailored education and long*-*term support* for both patient partners and other members of the research team to enable full participation, communication and engagement throughout all stages of the research process and beyond. Access to a developing evidence-base of good practice, or a registry of PE activities and initiatives, could support all researchers, including patient research partners, in developing their practice, skills and aptitudes to become effective PE practitioners. For example, experience from the OMERACT group highlighted the importance of developing a network of patient partners with a range of experiences who can offer ‘buddy’ support for newer and less experienced patient partners in the process [13, 19, 20]. However, as evidenced by groups such as OMERACT [13], EULAR [20] and PCORI [8], whilst such activities are essential to establish and maintain effective research partnerships, they are resource intensive—in terms of time and cost.

*Supportive institutions that value the contribution of PE* whilst the financial requirements of effective PE are increasingly recognised by grant awarding bodies, the lack of institutional support has been reported as a barrier to PE [4]. The additional, and often upfront, costs of PE throughout the research process—including the appropriate training of patient partners and researchers—must be recognised and appropriately budgeted for (http://www.pcori.org/sites/default/files/PCORI-Budgeting-for-Engagement-Activities.pdf). Supportive institutional policies, underpinned by reference to core PE values such as those established in this study, are essential when seeking to develop a strong and sustainable foundation for PE activities.

In providing the first, international and multi-perspective statement of values for PE in HRQoL, measurement science and PRO-related research, this study provides a strong foundation for the alignment of future PE activities and good patient engagement practice. The wider adoption of these values will facilitate a more transparent understanding of the importance of PE in this field.
